# Membrane Proteins Mediating Reception and Transduction in Chemosensory Neurons in Mosquitoes

**DOI:** 10.3389/fphys.2018.01309

**Published:** 2018-09-20

**Authors:** Jackson T. Sparks, Gina Botsko, Daniel R. Swale, Linda M. Boland, Shriraj S. Patel, Joseph C. Dickens

**Affiliations:** ^1^Biology Department, High Point University, High Point, NC, United States; ^2^Department of Entomology, Louisiana State University AgCenter, Baton Rouge, LA, United States; ^3^Department of Biology, University of Richmond, Richmond, VA, United States

**Keywords:** olfaction, taste, gustation, mosquito, insect, vertebrate, membrane proteins, ion channels

## Abstract

Mosquitoes use chemical cues to modulate important behaviors such as feeding, mating, and egg laying. The primary chemosensory organs comprising the paired antennae, maxillary palps and labial palps are adorned with porous sensilla that house primary sensory neurons. Dendrites of these neurons provide an interface between the chemical environment and higher order neuronal processing. Diverse proteins located on outer membranes interact with chemicals, ions, and soluble proteins outside the cell and within the lumen of sensilla. Here, we review the repertoire of chemosensory receptors and other membrane proteins involved in transduction and discuss the outlook for their functional characterization. We also provide a brief overview of select ion channels, their role in mammalian taste, and potential involvement in mosquito taste. These chemosensory proteins represent targets for the disruption of harmful biting behavior and disease transmission by mosquito vectors.

## Introduction

Mosquitoes are able to sense and track hosts over long distances, sometimes up to 70 m away (Chaisson and Hallem, 2012), a feat of chemosensitivity that increases the likelihood of encountering a host and transmitting disease. Mosquito chemosensation in adults includes two modalities – olfaction (smell) and gustation (taste), each crucial for host seeking, foraging, mating, and oviposition (Clements, 1992). Proper discrimination of chemical cues ensures a nutritive diet, suitable mates, and safe passage of genetic material to subsequent generations. Host-seeking behavior over a distance is informed by the olfactory system, while contact discrimination relies on the gustatory system. Overlap between olfactory and gustatory modalities is evident in some instances at both the anatomical and molecular levels (Melo et al., 2004; Kwon et al., 2006; Riabinina et al., 2016).

To locate human hosts, mosquitoes sense carbon dioxide (CO_2_), along with human skin and sweat odorants such as ammonia, lactic acid and other carboxylic acids (Chaisson and Hallem, 2012). Upon landing, skin emanations and blood are evaluated before full feeding behavior is initiated and completed. Disease agents are transmitted when saliva containing these agents passes into the circulatory system or epithelial tissue of the host via specialized mouthparts of the mosquito (Clements, 1992).

Transmission of malaria by mosquitoes led to an estimated 445,000 deaths world wide in 2016 (World Health Organization [WHO], 2017; Report 2017). Mosquito borne dengue virus is responsible for at least 22,000 deaths per year (Weaver and Reisen, 2010) and represents a worsening threat according to the World Health Organization. Cases of malaria and other mosquito borne diseases number in the hundreds of millions each year, representing one of the largest healthcare burdens in the world. Pathogen transfer between mosquitoes and humans is facilitated by highly efficient chemosensory neurons in the mosquitoes that guide them to their animal hosts. A full understanding of the chemosensory receptors and other membrane proteins that transduce the chemical signals responsible for guiding behavior is important to the development of strategies to disrupt host-seeking and biting by mosquito vectors.

Here we detail the repertoire of peripheral ligand binding membrane proteins, ancillary membrane proteins, and signal transduction proteins, and discuss the outlook for their functional characterization. These chemosensory proteins are the primary molecular detectors of ecologically relevant chemicals and as such represent targets for disruption of mosquito behavior for prevention of dangerous contacts by mosquito vectors with their hosts. We restrict our review to the adult stage, but the gene families highlighted also express in mosquito larvae and may be involved with behavior of aquatic life stages (Bohbot et al., 2007; Xia et al., 2008). We focus on information gathered from mosquito species; however, the broader context of these genes requires consideration of functional data from other insect families and model organisms.

## Chemosensory Anatomy

**FIGURE 1:**
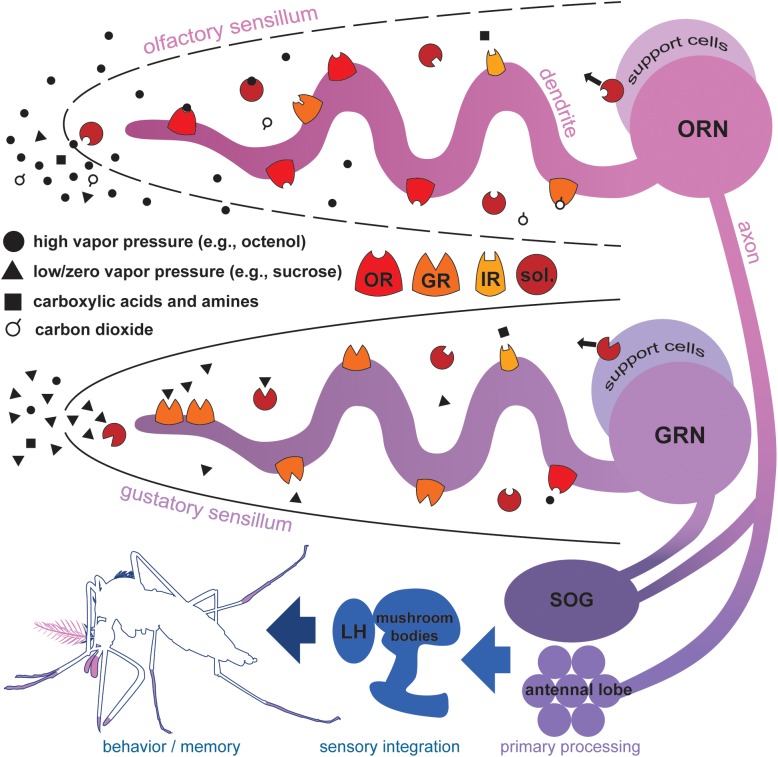
Proteins mediating chemosensation in mosquitoes. Molecules with high vapor pressures (solid circles and open circles), and volatile carboxylic acids and amines (black squares) contact aqueous sensillar lymph via cuticular pores in olfactory sensilla of the antennae and maxillary palps. Soluble proteins (sol.) secreted by support cells selectively shuttle molecules to dendritic processes of odorant receptor neurons (ORNs). Odorant receptors (OR), ionotropic receptors (IR), and carbon dioxide-sensitive gustatory receptors (GR) on the membrane of ORNs selectively bind molecules initiating signal transduction leading to ORN activation. In general, ORs, IRs, and GRs do not co-express in the same ORN, but are shown together here to illustrate protein richness of ORN/lymph interface. Molecules with low or zero vapor pressures (black triangles), acids and amines contact aqueous sensillar lymph via a terminal cuticular pore in gustatory sensilla of the labella, tarsi, and wing margins. Soluble proteins secreted by support cells selectively shuttle molecules to dendritic processes of gustatory receptor neurons (GRN). GRs, IRs, and some ORs on the membrane of GRNs selectively bind molecules initiating signal transduction and GRN activation. ORN and GRN axons terminate in the antennal lobes and subesophageal ganglion (SOG). Local interneurons mediate primary processing of chemosensory information, and signals project via second order projection neurons to higher brain regions associated with the mushroom bodies and lateral horn (LH) (Siju et al., 2008) where sensory information integrates subsequently informing important behaviors and shaping memory (Zars, 2000).

Chemosensory organs of mosquitoes include external paired antennae, maxillary palps, labial palps, internal surfaces of mouthparts, distal leg segments and wing margins that are adorned with hair-like or dome shaped structures called sensilla (Slifer, 1962). The morphology of individual sensilla varies by species and cuticular location. Sensilla have one or multiple pores that allow external molecules to traverse an aqueous lumen that is innervated by the dendrites of one or more olfactory receptor neurons (ORNs) or gustatory receptor neurons (GRNs) (**Figure 1**; Clements, 1992). These dendrites contain membrane-bound chemosensory receptor proteins that respond with sensitivity and selectivity to chemicals that pass into the lumen of the sensillum (Hallem et al., 2004). The interactions between molecules and receptor proteins initiate signal transduction leading to an action potential. This conversion of chemical information to electrical signals allows mosquitoes to detect individual components of complex blends providing the basis for higher neural processing in the antennal lobes, mushroom bodies and elsewhere in the brain. These chemical signals may be used to locate and identify food sources, oviposition substrates, conspecifics, and potential threats (Brown et al., 1951; Davis, 1984).

Water-soluble accessory proteins, including odorant binding proteins (OBPs), of the lumen originate from support cells near the cell body of ORNs and GRNs (**Figure 1**; Vogt and Riddiford, 1981; Vogt et al., 1999). These proteins have various functions in insects including transport of odorants and tastants to dendritic interfaces and general maintenance of the biochemical content of the sensillum (Vogt and Riddiford, 1981; Leal, 2013; Jeong et al., 2013). The binding profiles and exact roles of individual OBPs associated with ORN/GRN activity of mosquitoes remains mostly unexplored. Fan et al. (2011), Brito et al. (2016), and Pelosi et al. (2018) provide comprehensive reviews of insect OBPs. Mosquitoes present two or three support cells per sensillum; these cells sheath and maintain the proper function of sensory neurons (McIver, 1982). Axons of ORNs and GRNs project as nerve bundles to organized neuropil in the brain. In general, ORN termini are more distinctly organized than termini of GRNs in insects. In the malaria mosquito *Anopheles gambiae*, ORN termini group by subtype in 60–70 visible glomeruli in the antennal lobe and four to six less defined glomeruli in the subesophageal ganglion (Riabinina et al., 2016). GRNs of the yellow fever mosquito *Aedes aegypti* terminate into seven irregular zones in the subesophageal ganglion and tritocerebrum (Ignell and Hansson, 2005); these divisions may represent different classes of molecules stimulating each grouping of GRNs, e.g., sugars or human sweat components, as observed in the vinegar fly *Drosophila melanogaster* (Isono and Morita, 2010).

## Primary Receptor Families

**FIGURE 2:**
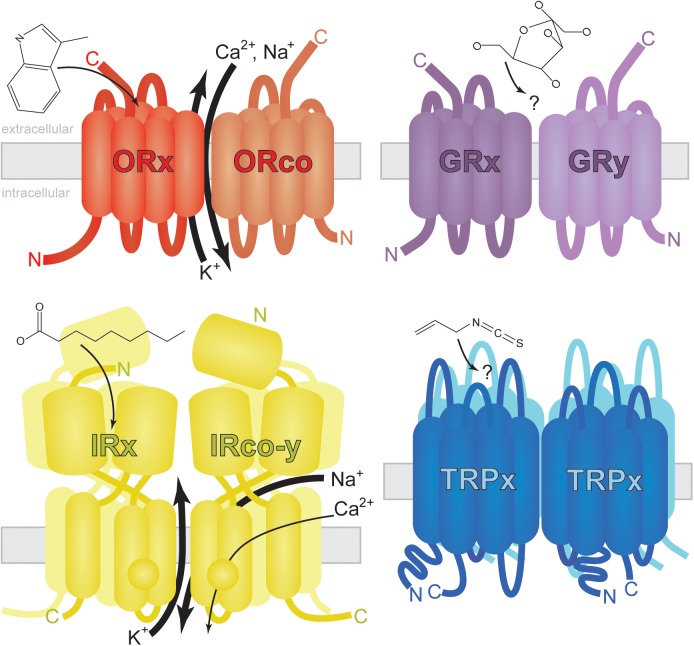
Generalized structure and membrane topology of insect chemosensory receptors. Odorant Receptors (ORs) are arranged with carboxy-termini (C) outside dendritic membranes where odorants (skatole shown as example) or odorant/soluble protein complexes activate ionotropic and/or metabotropic signal transduction (reviewed by Wicher, 2015). The exact nature and location of ion exchange is controversial and may vary by insect species. Odorants interact with the ORx hetero partner (small arrow) which facilitates the fast exchange of calcium, sodium and potassium cations (large arrows) via a ubiquitous coreceptor ORco. A slower G-protein-mediated response has been observed and may be affected by sub threshold concentrations of odorant (Wicher et al., 2008). Gustatory Receptors (GRs) share the same membrane topology with ORs (Zhang et al., 2011). Tastants (glucose shown as example) activate GR heterodimers, homodimers, or multimers leading to cell depolarization (Fujii et al., 2015). Little is known about GR-mediated signal transduction as heterologous expression of GRs has been largely unsuccessful (see Sato et al., 2011 for exception). Ionotropic Receptors (IRs) may function as dimers of heterodimers or in other trimeric conformations (Abuin et al., 2011). Carboxylic acids (nonanoic acid shown as example) and amines/imines may interact with ligand binding IRs (IRx) in mosquitoes (Pitts et al., 2017) facilitating the exchange of sodium and potassium ions as well as low levels of calcium ions via secondary channel activity (Abuin et al., 2011). Presumptive IR coreceptors (IRco-y) are required for the reception of multiple chemical classes in multiple cell types (Abuin et al., 2011; Pitts et al., 2017), but their exact role in signal transduction remains elusive. Transient Receptor Potential (TRP) channels assemble as homotetramers (Survery et al., 2016) sensitive to electrophiles (allyl isothiocyanate shown as example). These multimodal channels are approximately three times as large as ORs and GRs.

The three main chemosensory receptor families expressing in mosquito appendages containing ORNs/GRNs are the odorant receptors (ORs), ionotropic receptors (IRs), and gustatory receptors (GRs) (Pitts et al., 2004, 2011; Bohbot et al., 2007; Sparks et al., 2014; Lombardo et al., 2017). The expression of these gene families has been demonstrated in more than ten mosquito species belonging to the three most important disease spreading genera: *Aedes, Anopheles*, and *Culex*. GRs represent the most ancient insect chemosensory receptor protein family (Eyun et al., 2017), dating back to the most recent common ancestor of hexapods and placozoans, multicellular animals with the simplest known cellular structure. GR genes are present in diverse aquatic animals from anemones to copepods (Robertson, 2015; Eyun et al., 2017), perhaps mediating reception of water-soluble molecules. GRs and the more recently evolved, hexapod-specific ORs are related protein families (Eyun et al., 2017), each family with characteristic seven-transmembrane structure and atypical membrane topologies (**Figure 2**; Robertson et al., 2003). OR genes are present in wingless hexapod ancestors of insects belonging to Archaeognatha and Zygentoma but are absent in more ancient hexapod lineages (Brand et al., 2018). OR gene families expanded around the time of the first winged insects, perhaps as an adaptation to navigating larger areas with more diverse and informative odorants (Missbach et al., 2014). After adapting to life on land, but before the evolution of flight, IRs and the first ORs likely mediated reception of volatile odorants in early insect ancestors. As they predate ORs, IRs are present in multiple phyla of protostomes including molluscs, nematodes, and arthropods (Eyun et al., 2017).

The number of receptor genes in mosquitoes varies depending on species and gene family (**Table 1**), likely reflecting the unique requirements of each species’ ecological niche. Insect ORs are sensitive to compounds like esters, alcohols, and ketones, while IRs respond to various amines and acids (Suh et al., 2014). Comparative studies of receptor function are limited, but evidence suggests that relatively high sequence homology between a few mosquito ORs indicates conservation of an ancient and indispensable olfactory sensitivity to indole (Bohbot et al., 2010) and octenol, important cues for oviposition and host orientation (Dekel et al., 2016). The mechanism by which new receptor genes evolve may be primarily through a birth-and-death model wherein duplications lead to subtle fitness cost-free shifts in receptor shape/function (Robertson et al., 2003; Hill et al., 2015). There is also evidence that ORs lacking close sequence homology between two distantly related mosquito species each respond to the same human skin odor sulcatone (Bohbot et al., 2007; Carey et al., 2010), suggesting that shared host preferences of distant relatives may be the product of independent evolution of similarly sensitive ORs. The most highly conserved receptor genes across mosquito lineages retaining functions and/or expression profiles are the CO_2_- (Erdelyan et al., 2012; McMeniman et al., 2014) and sugar-sensitive GRs (Freeman et al., 2014), OR co-receptor (Jones et al., 2005), and the presumptive IR co-receptors (Rytz et al., 2013). As the majority of mosquito chemosensory receptors are highly divergent, functional characterization will require species-specific gene disruption or heterologous expression studies.

**Table 1:** Number of gustatory receptor, olfactory receptor, and ionotropic receptor genes in four mosquito species spanning all major clades.

Species	Gustatory receptors	Odorant receptors	Ionotropic receptors
*Anopheles gambiae*	76^a^	79^c^	46^h^
*Culex quinquefasciatus*	123^d^	180^d^	69^h^
*Aedes aegypti*	91^a^	100^b^	95^h^
*Aedes albopictus*	≥30^e∗^	158^g^	102^f^

*Estimations are from genomic analyses except where indicated. Variations in gene number likely reflect unique selective pressures on each species over time, but the possibility of stochastic processes affecting gene number should be considered. ^a^Kent et al., 2008; ^b^Bohbot et al., 2007; ^c^Hill et al., 2002; ^d^Arensburger et al., 2010; ^e^Lombardo et al., 2017; ^f^Chen et al., 2017; ^g^Chen et al., 2015; ^h^Croset et al., 2010; ^∗^transcriptomic data only.*

Expression levels of individual receptors offer some insights into function. Changes in transcript levels of receptor genes in *A. gambiae* are correlated with moderate chemosensory neuron sensitivity shifts following a blood meal (Rinker et al., 2013a). In addition to expression shifts due to feeding state changes, there may be natural fluctuations in chemosensory protein abundance based on time of day (Rund et al., 2013; De Das et al., 2018). Mosquito feeding often peaks at dawn or dusk (Clements, 1992); thus, there may exist a relationship between functional demands for chemosensory proteins and temporal regulation of gene expression in peripheral neurons. *A. aegypti* display concurrent increases in ORN sensitivity to CO_2_ and octenol, and expression levels of corresponding *OR* and *GR* transcripts throughout their first 10 days of adulthood (Bohbot et al., 2013). Differential vectorial capacity between two closely related anopheline species may be defined in part by differential expression of olfactory receptor genes (Rinker et al., 2013b), and host preference differences between two *A. aegypti* subspecies are directly linked to expression differences of a single *OR* (McBride et al., 2014). Moreover, viral infection alters expression levels of *OR*s and *GR*s in antennae of *A. aegypti* (Sim et al., 2012), raising the possibility that infectious agents may have evolved the ability to promote host-seeking behavior in infected vectors by targeting transcriptional activation factors for chemosensory genes in mosquito cells.

### Gustatory Receptors (GRs)

The architecture of the insect gustatory system has been widely studied from the molecular to the organismal level. *GR*s are primarily expressed in proboscis, legs (Hill et al., 2002; Sparks et al., 2013; Matthews et al., 2016) and maxillary palps (Erdelyan et al., 2012; Bohbot et al., 2014). Though only a single *GR* gene knockout/knockdown study has been published for mosquitoes, GRs likely mediate gustatory reception in GRNs based on: (1) the requirement of GRs for normal responses to a variety of tastants in *D. melanogaster* (reviewed in Isono and Morita, 2010) and (2) their enriched expression in mosquito tissues containing the greatest number of GRNs (Sparks et al., 2013; Matthews et al., 2016; Lombardo et al., 2017). GRNs respond to salt, feeding stimulants (e.g., sugar), water, host blood components and feeding deterrents (e.g., quinine and DEET) (Pappas and Larsen, 1978; Kessler et al., 2013; Sanford et al., 2013; Sparks and Dickens, 2016). Functional studies of mosquito GRs are unavailable, with the exception of RNAi- and ZFN-based confirmations that two to three atypical GRs expressing in ORNs are required for the detection of CO_2_ in *A. aegypti* (Erdelyan et al., 2012; McMeniman et al., 2014). Direct investigation of specific insect GRs using heterologous systems has been reported for a single sugar sensitive receptor (Sato et al., 2011). Other attempts to express functional non-sugar-sensitive GR assemblages have been unsuccessful, thus the generation of GR mutant strains via CRISPR-mediated alterations will likely be the next step toward GR deorphanization. Several mosquito GRs show clear homology with *D. melanogaster* GRs of known function (Kent et al., 2008), namely those involved in the reception of sugars or antifeedants like quinine. Whether or not mosquito GRs play a role in the reception of host cues with low vapor pressures remains an intriguing possibility.

### Odorant Receptors (ORs)

Odorant receptors are expressed in the main olfactory appendages (Qiu et al., 2004): antennae, maxillary palps, and proboscis (Fox et al., 2001; Kwon et al., 2006; Lu et al., 2007). The best characterized chemosensory gene family in mosquitoes, ORs are required for normal host discrimination (DeGennaro et al., 2013) and the reception of important host cues (McBride et al., 2014). Components of human sweat (Bernier et al., 2000) activating *A. gambiae* ORNs include L-lactic-acid, l–octen–3–ol and 4–methylphenol (Cork and Park, 1996). Other host odorants known to stimulate mosquito ORNs include ammonia, indole, geranyl acetone, 3-methyl-1-butanol, 6-methyl-5-hepten-2-one, 1-dodecanol, hexanedioic acid (Meijerink et al., 2001; Bohbot et al., 2010; Pelletier et al., 2010), and skatole (Hughes et al., 2010). ORs are amenable to heterologous expression and subsequent chemical screening. The odorant tuning range of individual ORs varies greatly from narrow to broad (Carey et al., 2010; Wang et al., 2010). Some ORs are only activated by compounds within a single chemical class, e.g., *A. gambiae* OR2 is tuned to a small set of aromatics containing a benzene ring, while others respond to chemicals from multiple classes from terpenes to heterocyclic compounds (Carey et al., 2010; Wang et al., 2010).

DeGennaro et al. (2013) examined the relative contributions of ORs, GRs, and IRs to host-seeking behavior in *A. aegypti* by genomic deletion of the gene coding the OR-coreceptor (ORco, necessary for all OR-mediated ORN activation). In the absence of CO_2_, *ORco* mutants did not respond to human-scented materials as is the case for wildtype controls (DeGennaro et al., 2013), indicating the ORco-independent IRs are likely not involved in the detection of host skin emanations. However, in combination with CO_2_, which activates a unique set of GRs (Erdelyan et al., 2012), human skin odorants do indeed elicit behavioral responses from *ORco* mutants suggesting the existence of redundant OR-independent pathways for detecting blends of host breath and skin emanations.

McBride et al. (2014) compared antennal transcriptomes of human-preferring, domestic forms of *A. aegypti* with guinea pig-preferring forest forms thereby identifying the enriched transcript OR4 among 13 other genes as significantly upregulated in domestic forms and human-preferring hybrids. Not only does increased OR4 expression appear to drive human host preference in wild populations, but specific non-synonymous variants also show strong correlation to preference and demonstrate linear functional variance. OR4 is sensitive to sulcatone, a chemical found in uniquely high levels in human emanations as compared to other animals (McBride et al., 2014). Interestingly, levels of sulcatone exceeding those naturally emanating from human skin may elicit avoidance responses from *A. aegypti* (Logan et al., 2008). McBride et al. (2014) note that other odors besides sulcatone likely contribute to human preference by domestic forms of *A. aegypti* and other up- or down-regulated genes identified in their survey likely contribute to host preference.

ORs (Liu et al., 2010; DeGennaro et al., 2013; Xu et al., 2014) and GRs (Lee et al., 2010; Sanford et al., 2013) are involved with the reception of repellents like DEET. Xu et al. (2014) identified in the southern house mosquito *Culex quinquefasciatus* OR136 that, in combination with ORco, mediates responses to synthetic and natural repellents in *Xenopus* oocytes. Knockdown of *C. quinquefasciatus* OR136 transcripts reduced ORN responses to DEET (Xu et al., 2014). Repellents like DEET may also alter feeding and host seeking behaviors via interactions with many receptors at once, modulate ORco function directly or function primarily in coordination with other behaviorally relevant compounds like those emanating from host skin or breath (Dickens and Bohbot, 2013; DeGennaro, 2015).

### Ionotropic Receptors (IRs)

Ionotropic receptor expression in olfactory and gustatory organs in *D. melanogaster* is well characterized, and these receptors are tuned to carboxylic acids, aldehydes, and amines (Benton et al., 2009; Abuin et al., 2011). Acids and amines are important host-seeking signals for mosquitoes (Van der Goes van Naters and Carlson, 2006). Ligands of IR-expressing ORNs were originally identified through extracellular recordings of electrical activity of sensory neurons housed within target sensilla (Yao et al., 2005). IR-expressing neurons housed within grooved-peg sensilla of the antenna (Pitts et al., 2004) are much less sensitive and slower to respond than OR-expressing neurons in insects. In further contrast, IR and OR-expressing neurons detect different classes of odorants; the strongest IR ligands only weakly activate, if at all, ORs, and the strongest OR ligands (ester, alcohols, and ketones) do not stimulate IR-expressing neurons (De Bruyne et al., 2001).

Tuning profiles of individual IRs in mosquitoes is limited to two studies and a handful of individual genes (Liu et al., 2010; Pitts et al., 2017). A study implementing knock-down of *IR76b* in *A. gambiae* larvae demonstrated its function in mediating behavioral responses to butylamine (Liu et al., 2010). Pitts et al. (2017) is the only study to date examining individual IR gene function in adult mosquitoes and is consistent with foundational work in *Drosophila* (Benton et al., 2009; Croset et al., 2010; Abuin et al., 2011). Different combinations of *A. gambiae* IRs were expressed heterologously in *Xenopus* oocytes and more than 400 chemicals were used to screen for IR-dependent currents (Pitts et al., 2017). Three IR “complexes” were discovered: *IR41a/IR25a/IR76b* (most sensitive to nitrogenous compounds 2-methyl-2-thiazoline and pyrrolidine), *IR41c/IR25a/IR76b* (most sensitive to pyrrolidine and 3-pyrroline), and *IR75k/IR8a* (most sensitive to carboxylic acids of eight or nine carbons). Many other IR genes are expressed in mosquito chemosensory tissues (**Table 1**); thus, IR deorphanization represents a crucial step toward exploring all potential receptors as targets for altering harmful host-seeking and feeding behaviors.

## Signal Transduction

Ion channels serve as the molecular basis for membrane excitability by allowing inward or outward flow of ions across a cell membrane to enable signal transduction and the alteration of other cellular processes. Ligand-gated ion channels represent the primary ion channel type in the insect chemosensory system functioning as a “receptor.” ORs, GRs, and IRs act through synaptic signaling on electrically excitable cells by converting chemical signals (i.e., tastants or odorants) to an electrical signal. Upon binding of the signal molecule(s) several actions may allow flow of cations and/or anions that stimulate neuronal transmission, downstream signaling, and other physiological processes: the ion channel protein itself may open due to a conformational shift, associated ion channels may be activated in conjunction with ligand-binding receptor activation or intracellular modulators of channel activity may initiate transmembrane ion flow indirectly.

Stimulus-specific ORs (ssORs) in insects are trafficked to dendritic membranes by co-receptor ORco (Larsson et al., 2004), a membrane protein highly conserved in sequence and function across diverse insect lineages (Jones et al., 2005) and required for fast ssOR activation (Larsson et al., 2004). ORco and ssORs form heteromeric complexes acting as ligand-gated ion channels with evidence pointing toward a pore region shared between subunits (**Figure 2**; Sato et al., 2008; Wicher et al., 2008; Nichols et al., 2011; Nakagawa et al., 2012). Evidence based on Dipteran (*D. melanogaster* and *A. gambiae*) and Lepidopteran (*Bombyx mori*) OR complexes expressed in *Xenopus* oocytes and cultured human cells suggests G-protein-coupled pathways are dispensable for OR activation in insects (Sato et al., 2008). However, other experiments probing *D. melanogaster* OR complexes showed that G-protein modulating compounds or genetic disruption of G-proteins significantly affected OR activation dynamics in cultured human cells (Wicher et al., 2008; Deng et al., 2011) and *in vivo* (Deng et al., 2011). A more recent study found no evidence for ionotropic mechanisms in lepidopteran OR complexes sensitive to pheromone (Nolte et al., 2016). Thus, OR-mediated signal transduction in mosquitoes may involve ionotropic and/or metabotropic pathways. For in-depth reviews of olfactory transduction mechanisms in insects, see Fleischer et al. (2018) and Wicher (2015).

Co-receptor IRs (IRcos), such as *D. melanogaster* and *A. gambiae* IR25a and IR8a, form heteromeric relationships with stimulus-specific IRs (ssIRs) (Abuin et al., 2011; Pitts et al., 2017). IRcos are more conserved between insect species than ssIRs and possess an amino-terminal domain (ATD) that is usually absent in ssIRs (**Figure 2**). Evidence suggests IRs assemble as heterotetramers comprising two ssIR and two IRco subunits (Abuin et al., 2011; Pitts et al., 2017). Additional IR assemblies may exist as some combination of three IRs, including a second type of receptor (reviewed in Rytz et al., 2013). It is unknown whether these functional relationships apply to all mosquito IRs.

Gustatory receptor-mediated signal transduction remains poorly understood. Heterologous expression of gustatory GRs has not been as successful as similar experiments using ORs and IRs, perhaps meaning that either many GRs are required simultaneously to produce single cell responses or that other unknown factors present in GRNs are required for ligand-gated activation. Orthologs *D. melanogaster* GR43b and *B. mori* GR9 act as fructose-sensitive non-selective ionotropic channels when expressed in *Xenopus* oocytes or cultured human cells (Sato et al., 2011). This activity was independent of G-protein-coupled pathways, though several other reports provide evidence that G-protein-coupled pathways are involved in GR-mediated signal transduction (Ishimoto et al., 2005; Ueno et al., 2006; Ueno and Kidokoro, 2008).

## Ancillary Membrane Proteins

As reviewed above, the majority of recent research concerning the molecular biology of mosquito chemoreception has focused on the function of three receptor families (ORs, GRs, and IRs), their ligands, phylogenetic analyses, and modulation of these receptors to obtain a desirable phenotype (i.e., avoidance). Recently, studies have defined the properties of GRNs and have elegantly shown functionally different classes of GRNs expressing unique combinations of receptor genes (Freeman and Dahanukar, 2015). Specifically, ion channels are expressed within various GRNs where they are required for relevant taste modalities, such as salt taste and bitter detection (Liu et al., 2003; Al-Anzi et al., 2006), as well as neural propagation of the signal.

### Transient Receptor Potential (TRP) Channels

Transient Receptor Potential (TRP) channels belong to the group of non-voltage gated, cation-permeable ion channels (Nilius, 2003) and are highly conserved proteins that are present in all species from yeast to mammals. Mammalian TRP channels are composed of six-transmembrane domains with a pore region between TM5 and TM6 (**Figure 2**) and can be divided into six subfamilies based on their sequence homology: TRPC (canonical), TRPV (vanilloid), TRPM (melastatin), TRPP (polycystin), TRPML (mucolipin), and TRPA (ankyrin) (Vannier et al., 1998). TRP channels respond to a wide range of stimuli and have an astonishing diversity of cation selectivity, which enables them to function as a conserved unit for integration of varied sensory information.

TRPA channels are a conserved subfamily of cation channels that are expressed in vertebrates and invertebrates, and appear to perform similar physiological functions. In vertebrates, the calcium permeable cation channel, TRPA1, is expressed in nociceptive neurons and functions to detect noxious or pungent chemicals, such as environmental irritants (Bautista et al., 2005). The majority of information regarding the physiological importance of insect TRP channels is focused on their role in the mechanisms of thermosensation and mechanosensation (reviewed in Fowler and Montell, 2013), yet over the past decade, TRPA channels have been of significant interest to insect physiologists for their role in gustation (Du et al., 2015; Freeman and Dahanukar, 2015) and repellency (Bautista et al., 2005; Kim et al., 2010; Kwon et al., 2010).

TRPA channels in *D. melanogaster* are encoded by *painless*, the fly homolog to mammalian TRPA1/ANKTM1 ion channel protein. Like GRs, *painless* is expressed in GRNs of the labellum, pharynx, legs and wings, and are specifically involved in the rejection of allyl and benzyl isothiocyanate, the pungent taste and insecticidal component of wasabi (Al-Anzi et al., 2006). TRPA1 is expressed in a subset of aversive GRNs and is required for avoiding aristolochic acid in food-choice assays (Kim et al., 2010), but avoidance of other bitter or aversive compounds, such as caffeine or quinine, were independent of TRPA1 function. This lack of broad activity to all bitter molecules suggests TRPA1 likely functions in tandem with additional transport proteins or receptors that may be differentially expressed. Indeed, a subset of labellar and leg GRNs coexpress the caffeine receptors (*Gr66a*, *Gr32a*, and *Gr47a*) and *painless*. The functional dependency of these two genes is not fully understood, but we speculate that the co-expression of these two receptors, and potentially others, enables a multimodal response neuron that can detect and integrate taste modalities that result in different behavior (Van Giesen et al., 2016).

Expression of *A. gambiae* TRPA1 (AgTRPA1) in *Xenopus* oocytes indicated that the channel transduces temperature sensation, and channel expression is on the distal antennal sensory structures (Wang et al., 2009). These structural and functional roles seem to be conserved in *A. aegypti* and the common house mosquito *Culex pipiens*. Together, these findings support the notion that *Ag*TRPA1 functions as a peripheral thermoreceptor in mosquito antenna. More recent work has uncovered an additional chemosensory role for *Ag*TRPA1 (Survery et al., 2016). Patch clamp recordings on heterologously expressed and purified, full-length *Ag*TRPA1 and truncated Δ1–776 *Ag*TRPA1 (lacking the N-terminal ARD) demonstrated that both proteins are functional, as each responded to the electrophilic compounds, allyl isothiocyanate and cinnamaldehyde, as well as heat. Their similar intrinsic fluorescence properties and related quenching of tryptophan, when activated by allyl isothiocyanate or heat, led the researchers to conclude that conformational change in the lipid bilayer occurs independently and outside of the N-terminal domain (Survery et al., 2016). As such, *Ag*TRPA1 is both a thermo- and chemoreceptor, and while the N-terminal domain’s function is unknown, it is hypothesized to play a role in tuning the channel’s response (Survery et al., 2016).

Kang et al. (2010) examined the response of *D. melanogaster* to reactive electrophiles, including allyl isothiocyanate (AITC), N-methyl maleimide (NMM), and cinnamaldehyde (CA), and found that addition of these chemicals to food dramatically inhibited the natural proboscis extension response (PER); the inhibitory effect was considered gustatory, not olfactory, because avoidance of these non-volatiles required ingestion. This study found the responses to reactive electrophiles depend on the cation channel TRPA1 as TRPA1 mutants showed no reduction in PER when offered food containing AITC, NMM, or CA. Promoter-knockdown experiments established peripheral sensory neurons as the site of action for dTRPA1 in gustation. TEVC recordings on *A. gambiae* demonstrate that reactive electrophiles activate mammalian TRPA1s; mutations in TRPA1 decreased electrophile sensitivity.

Citronellal, a plant-based acyclic monoterpene with a distinctive lemony scent is used in lotions, candles, and sprays to repel mosquitoes and other pests such as ticks and fleas. In contrast to *D. melanogaster* for which citronellal activated a GPCR coupled to TRPA1 channels, *A. gambiae* TRPA1 was directly activated by citronellal (Kwon et al., 2010). These results invite further study to confirm the potential of repellents like citronellal that activate gustatory signaling in mosquitoes and secondarily deter feeding. *A. gambiae* isoform TRPA1(B) did not respond robustly to citronellal (Du et al., 2015). These results encourage a comparative structure-function approach using TRPA1(A) and TRPA1(B) to probe the structural basis for citronellal actions on the cation channel activity.

TRPA1 channels are not present in hymenoptera (although they do have other TRPA channels; Matsuura et al., 2009). This suggests that TRPA1 could be targeted for mosquito control without negatively impacting important pollinators like honeybees. Further, the gustatory system of *D. melanogaster* employs TRPA1 for detection and subsequent avoidance of bacterial endotoxins lipopolysaccharides (LPS) (Soldano et al., 2016). Together, these data support the notion that TRPA channels are a highly conserved ion channel and have similar physiological roles in the sensory systems of mammals and invertebrates, regardless of the sensory modality involved (Rosenzweig et al., 2005).

In addition to TRPA channels, other TRP channels play key roles in gustatory avoidance. For instance, TRPL, a member of the TRPC family (Venkatachalam and Montell, 2007), is activated *in vitro* by the bitter tastant camphor and is expressed in the dendrites of *D. melanogaster* GRNs (Zhang et al., 2013). Wildtype adult and larval *D. melanogaster* avoid camphor, whereas TRPL302 mutants displayed a deficit in camphor avoidance while showing normal avoidance in response to other aversive tastants (Zhang et al., 2013). Interestingly, TRPL expression was reduced during prolonged exposure to camphor, and a corresponding reduction in avoidance behavior was observed (Zhang et al., 2013). These data suggest that changes in taste preference are dependent upon the concentration of receptors, and modifications of synaptic connections or receptor concentration may result in plasticity of taste interpretation; thus, this pathway may represent a novel target for antifeedants for arthropod control.

### Epithelial Sodium Channels (ENaC)

Epithelial Sodium Channels (ENaC), a member of the degenerin (DEG)/ENaC superfamily of ion channels encoded by the pickpocket (*ppk*) gene family, functionally assemble as a heterotrimeric or homotrimeric proteins (Benson et al., 2002). ENaC channels have evolved different physiological functions throughout the Kingdom Animalia, but are conserved as ionotropic receptors that respond to extracellular stimuli to pass sodium ions.

Although insects and mammals have independently evolved distinct molecular pathways for gustation, there are clear parallels in the molecular organization that allows for comparison between the two systems (Yarmolinsky et al., 2009). In mammals, ENaC is involved in transepithelial sodium transport in many tissues (e.g., kidney, lung) and is critical in many epithelial tissues that require sodium transport, including taste epithelial cells (Lindemann et al., 1998; Kretz et al., 1999). Genetic knockout of ENaC in rat taste cells resulted in loss of salt attraction and sodium taste response, which validated previous pharmacological studies suggesting ENaC as the principal pathway for mediating sodium taste in mammals (Lindemann, 1997; Chandrashekar et al., 2010). Interestingly, significant overlap in behavior exists between insects and mammals when exposed to varying concentrations of salts. Considering the conservation of ENaC function and similar behavioral tendencies, it was hypothesized that DEG/ENaC proteins are responsible for salt detection in *D. melanogaster*. Indeed, two genes encoding ENaC, termed *Pickpocket11* (*ppk11*) and *Pickpocket19* (*ppk19*), are expressed in the taste-sensing terminal organ of larvae and in the taste bristles of the labella, legs, and wing margins of adult flies (Zelle et al., 2013). Importantly, knockdown of *ppk11* and *ppk19* resulted in loss of behavioral and electrophysiological responses to low salt concentrations. Similarly, disrupting *ppk11* or *ppk19* in adults negatively affected the response to high salt concentrations by eliminating avoidance behavior (Liu et al., 2003). The authors concluded that the DEG/ENaC channels encoded by *ppk11* and *ppk19* are critical to the detection of Na^+^ and K^+^ salts and contribute to the behavioral responses to various salt concentrations.

Analysis of the *A. gambiae* genome revealed that the *ppk* gene family members were reduced when compared to *D. melanogaster* with the mosquito consisting of 18 family members, of which 17 had homologs in the *D. melanogaster* genome which contains 31 total *ppk* genes (Zelle et al., 2013). Importantly, subfamily III of the *Drosophila* ENaC gene family (containing *ppk19*) was absent in the *A. gambiae* genome which suggests mosquitoes may use a different *ppk* gene product to detect salt.

In addition to salt detection, DEG/ENaC channels are responsible for mediating activity of water-sensitive GRNs in insects. The *D. melanogaster* gene *ppk28* encodes a DEG/ENaC channel that is osmo-sensitive and is expressed in the taste bristles, but not in taste pegs, which was correlated back to a water-sensing neuron through imaging of an enhancer-trap Gal4 line (Cameron et al., 2010). To test the functional role of *ppk28*, the authors generated a *ppk28* null mutant and performed extracellular bristle recordings of l-type labellar sensilla. Recordings showed the mutant cells were completely insensitive to water, but were equally sensitive to sucrose when compared to controls (Cameron et al., 2010). The localization and functional data suggest *ppk28* encodes an ENaC channel that responds to low osmolarity to mediate both GRN and behavioral responses to water. While it is evident that *ppk* gene products are part of the physiological cascade to detect water, it is likely that ENaC is functionally coupled to a series of transporters. For instance, water transport channels, such as aquaporins, are expressed at the apical and basolateral membranes of rat taste cells and are critical for the gustatory response to water in mammals (Watson et al., 2007).

Gene products from the *ppk* family appear to be functionally conserved from mammals to insects and are responsible for detection of Na^+^ and K^+^ salts. Less is known regarding the functional conservation of their role as a sensor of osmolarity between insects and mammals. Similarly, the role of additional membrane transport pathways, such as aquaporins, and the interaction of these proteins with *ppk* gene products for tasting salt and sensing water remains to be determined. In summary, like TRP channel genes, *ppk* genes are potential gateways to activate avoidance behavior as transcripts of many conserved *ppk*s are abundant in the taste organs of *A. aegypti* (Sparks et al., 2014).

## Underexplored Ion Channels in Insect Gustatory Signaling

Taste cells are excitable cells that use a vast array of receptors and ion channels during their activity (Bigiani et al., 2002). In particular, taste cells are known to express a variety of voltage-sensitive ion channels, such as voltage gated (vg) sodium and potassium ion channels, that mediate the generation and/or propagation of action potentials (Herness and Sun, 1995; Chen et al., 1996; Ohmoto et al., 2006). The gustatory cells of mammals are known to have polarized epithelia with clear functional separation of apical and basolateral membranes (Purves et al., 2001). It is well established that proper function of any polarized epithelial tissue requires strict regulation and maintenance of the membrane potential and membrane resistance to enable an intracellular current that drives ion transport. Thus, it is reasonable to speculate that ion channels serve as gustatory receptors while also serving as critical components of the machinery responsible for proper polarization and conductance of GRNs.

The presence of these ion channels in mammalian taste cells combined with the conserved physiology of gustatory cells across organisms raises the intriguing possibility that these channels may also be functionally important for insect gustation. We provide a brief overview of select ion channels and their role in mammalian and insect taste systems below.

### Potassium (K^+^) Ion Channels

K^+^ ion channels are diverse, widespread, and have been detected in almost every eukaryotic cell type examined (Latorre and Miller, 1983; Rudy, 1988). These channels represent a fundamental component of animal physiology by establishing and maintaining the membrane potential of cells, which is required for nearly all cellular functions (Urrego et al., 2014). They also play critical roles in signal integration and some function to link metabolism or cell signaling to electrical activity. Yet, despite the functional relevance of K^+^ ion channels, the role of these channels in insect gustation and their potential utility are unexplored and ripe for discovery.

### Voltage-Gated K^+^ (vg-K^+^) Ion Channels

In mammalian taste cells, two vg-K^+^ channels, KCNQ1 and KCNH2, are expressed and involved in the repolarization of taste receptor cells. Interestingly, in one study the channels showed no specific taste modality (Ohmoto et al., 2006), which indicates these channels are likely involved in regulating the action potential. However, a study of rat fungiform taste receptors provided significant evidence that a vg-K^+^ channel was involved in the detection and preference of polyunsaturated fatty acid (PUFA) molecules (Gilbertson et al., 1997). A delayed outwardly rectifying potassium current was reversibly inhibited by extracellular application of arachidonic acid (C20:4) or linoleic acid (C18:2) in whole cell patch clamp recordings from taste cells. Further, the same study showed that PUFAs activated inwardly rectifying potassium (Kir) currents. vg-K^+^ channels regulate action potential firing and may be a target for taste stimuli (Kinnamon, 1992; Gilbertson, 1993), and Kir channels are important for establishing resting membrane potential and shunting current from the apical to basolateral membrane. Thus, a bimodal effect of PUFA on two distinct K^+^ channel types with opposing conductance directions suggests PUFA may prolong the stimulus-induced depolarization to amplify the signal and ensure neurotransmitter release from the basolateral region of the cell (Gilbertson et al., 1997). Considering this and because vg-K^+^ ion channels are exploitable insecticide targets (Bloomquist et al., 2014), the role of these channels should be studied in gustatory reception and signaling to enable a more holistic understanding of insect gustatory pathways and to test the deterrent nature of these channels for mosquito management.

### Inwardly Rectifying Potassium (Kir) Channels

Kir channels characterized from taste cells of rats are weak to moderate inward rectifiers (Sun and Herness, 1996) and contribute to both the resting and active states of the membrane potential (Hibino et al., 2010). In glial cells, Kir channels function as a route of K^+^ clearance in the central nervous system of mammals (Kofuji and Newman, 2004; Neusch et al., 2006). Similar to the central nervous system, repetitive firing of taste cells will result in elevation of extracellular potassium ions that requires homeostatic mechanisms to clear K^+^ ions from the extracellular space and distribute them back to areas of low intracellular K^+^ concentration gradient. Indeed, Kir1.1, or ROMK, is localized at the apical tip of rat taste cells above the apical tight junctions, and was speculated to function as a route for buffering K^+^ gradients during taste cell activity (Dvoryanchikov et al., 2009). Previous reports indicated that Kir channels are responsible for buffering K^+^ ion gradients during neural activity of *D. melanogaster* (Chen and Swale, 2018) in a near identical manner as for mammals. This conserved role of mammalian and *D. melanogaster* neural Kir channels in buffering K^+^ gradients in mammalian taste cells indicates these channels may serve a similar function in mosquito gustatory systems.

In addition to establishing K^+^ gradients during cell function, Kir channels mediate transduction for sour and sweet taste (Yee et al., 2011; Ye et al., 2016). The mechanism of sweet taste in mammals is not completely understood because knockout of the gene encoding the determinant of saccharin and sugar preference (T1r3) (Fuller, 1974) does eliminate the response to glucose or other sugars (Damak et al., 2003). Another type of Kir channel, ATP-gated Kir (K_ATP_) channels, which serve as metabolic sensors in a variety of mammalian cell types, were co-expressed in taste cells with sugar transporters and glucose sensor proteins (Yee et al., 2011). Based on electrophysiological studies that confirmed K_ATP_ channel current to be functional, it was concluded that these channels regulate taste sensitivity to sweet molecules according to metabolic needs (Yee et al., 2011). K_ATP_ channels are underexplored in insects when compared to mammals, even though these channels are critical for a variety of physiological functions in taxonomically diverse arthropods, such as innate antiviral immunity (Eleftherianos et al., 2011; O’Neal et al., 2017a), honeybee heart function (O’Neal et al., 2017b), salivary gland function and feeding (Swale et al., 2017). Future work should investigate the physiological role and toxicological relevance of Kir/K_ATP_ channels in mosquito gustation.

### Voltage-Gated Chloride (Cl^−^) Channels

Scant information exists regarding the expression patterns or physiological role of chloride (Cl^−^) channels in arthropod gustatory systems. However, previous work suggests that mammalian taste cells possess several types of Cl^−^ channels that play a key role in signal transduction of taste cells (Miyamoto et al., 1998; Herness and Sun, 1999). It was suggested that vg-Cl^−^ channels contribute to the membrane potential and electrical excitability but are not involved in the initiation of action potentials (Huang et al., 2005). Immunohistochemical studies show that ClC-4 and ClC-4A are expressed on the plasma membrane as well as intracellular membranes of taste cells (Zhang et al., 2013), suggesting a possible role in neurotransmitter uptake and regulation of synaptic activity. ClC-4A may also be a candidate Cl^−^ channel for acid transduction in sour taste by contributing to acidification of intracellular organelles (Huang et al., 2005). Also, ClC-3 is expressed in the synaptic vesicles of taste neurons and dissipates the membrane potential generated by the inevitable buildup of H^+^ by serving as an electrical shunt for vesicular acidification (Huang et al., 2005). While Cl^−^ channels are likely a component of sour taste, Kir2.1 may also function in tandem with a proton pump for sour taste transduction in mammals (Ye et al., 2016). Together, the results suggest that detection of sour taste is a complex process that remains to be fully elucidated. In addition to the studies on mammalian taste cells, mutation of a gene encoding a glutamate-gated chloride channel in the nematode *Caenorhabditis elegans* results in reduced gustatory plasticity. We suggest a need to explore this functional role of Cl^−^ channels in insect gustation.

## Concluding Remarks

A full understanding of how individual odorants, tastants, or blends are detected, converted to neural signals and processed will depend on determining the functional relationships between all chemosensory gene families and the cells expressing them. Characterizing the response profiles of receptor complexes and comparing these responses among diverse mosquito species will further our understanding of how these successful animals have filled so many ecological niches and rapidly adapted to host availability.

Recent advances in our ability to quickly and reliably edit target genes in the germline of mosquitoes should help uncover unknown roles of key molecular components of olfactory and gustatory tissues. These studies are tedious and costly, but clarity of function will help define convenient targets for the development of novel repellents and antifeedants. In addition to high-throughput chemical screens, sophisticated modeling and simulation software can be developed and used to discover the most likely compounds capable of selectively activating or blocking neural pathways associated with harmful mosquito behaviors. Central to the creation of new vector control strategies is achieving greater resolution of ORN/GRN/IR function and the interactions between receptor complexes, ion channels and host-derived ligands.

## Author Contributions

All authors listed have made a substantial, direct and intellectual contribution to the work, and approved it for publication.

## Conflict of Interest Statement

The authors declare that the research was conducted in the absence of any commercial or financial relationships that could be construed as a potential conflict of interest.

## References

[B1] Abuin L. Bargeton B. Ulbrich M. H. Isacoff E. Y. Kellenberger S. Benton R. (2011). Functional architecture of olfactory ionotropic glutamate receptors. *Neuron* 69 44–60. 10.1016/j.neuron.2010.11.042 21220098 PMC3050028

[B2] Al-Anzi B. Tracey W. D. Jr. Benzer S. (2006). Response of *Drosophila* to wasabi is mediated by painless, the fly homolog of mammalian TRPA1/ANKTM1. *Curr. Biol.* 16 1034–1040. 10.1016/j.cub.2006.04.002 16647259

[B3] Arensburger P. Megy K. Waterhouse R. M. Abrudan J. Amedeo P. Antelo B. (2010). Sequencing of *Culex quinquefasciatus* establishes a platform for mosquito comparative genomics. *Science* 330 86–88. 10.1126/science.1191864 20929810 PMC3740384

[B4] Bautista D. M. Movahed P. Hinman A. Axelsson H. E. Sterner O. Högestätt E. D. (2005). Pungent products from garlic activate the sensory ion channel TRPA1. *Proc. Natl. Acad. Sci. U.S.A.* 102 12248–12252. 10.1073/pnas.0505356102 16103371 PMC1189336

[B5] Benson C. J. Xie J. Wemmie J. A. Price M. P. Henss J. M. Welsh M. J. (2002). Heteromultimers of DEG/ENaC subunits form H+-gated channels in mouse sensory neurons. *Proc. Natl. Acad. Sci. U.S.A.* 99 2338–2343. 10.1073/pnas.032678399 11854527 PMC122366

[B6] Benton R. Vannice K. S. Gomez-Diaz C. Vosshall L. B. (2009). Variant ionotropic glutamate receptors as chemosensory receptors in *Drosophila*. *Cell* 136 149–162. 10.1016/j.cell.2008.12.001 19135896 PMC2709536

[B7] Bernier U. R. Kline D. L. Barnard D. R. Schreck C. E. Yost R. A. (2000). Analysis of human skin emanations by gas chromatography/mass spectrometry. 2. Identification of volatile compounds that are candidate attractants for the yellow fever mosquito (*Aedes aegypti*). *Anal. Chem.* 72 747–756. 10.1021/ac990963k 10701259

[B8] Bigiani A. Cristiani R. Fieni F. Ghiaroni V. Bagnoli P. Pietra P. (2002). Postnatal development of membrane excitability in taste cells of the mouse vallate papilla. *J. Neurosci.* 22 493–504. 10.1523/JNEUROSCI.22-02-00493.2002 11784795 PMC6758677

[B9] Bloomquist J. R. Mutunga J. M. Islam R. M. Verma A. Ma M. Totrov M. M. (2014). “Voltage-sensitive potassium Kv2 channels as new targets for insecticides,” in *Biopesticides: State of the Art and Future Opportunities*, ed. Gross A. D. (Washington, DC: Oxford University Press), 71–81.

[B10] Bohbot J. Pitts R. J. Kwon H. W. Reutzler M. Robertson H. M. Zwiebel L. J. (2007). Molecular characterization of the *Aedes aegypti* odorant receptor gene family. *Insect. Mol. Biol.* 16 525–537. 10.1111/j.1365-2583.2007.00748.x 17635615 PMC3100214

[B11] Bohbot J. D. Durand N. F. Vinyard B. T. Dickens J. C. (2013). Functional development of the octenol response in *Aedes aegypti*. *Front. Physiol.* 4:39. 10.3389/fphys.2013.00039 23471139 PMC3590643

[B12] Bohbot J. D. Jones P. L. Wang G. Pitts R. J. Pask G. M. Zwiebel L. J. (2010). Conservation of indole responsive odorant receptors in mosquitoes reveals an ancient olfactory trait. *Chem. Senses* 36 149–160. 10.1093/chemse/bjq105 20956733 PMC3020388

[B13] Bohbot J. D. Sparks J. T. Dickens J. C. (2014). The maxillary palp of *Aedes aegypti*, a model for multisensory integration. *Insect Biochem. Mol. Biol.* 48 29–39. 10.1016/j.ibmb.2014.02.007 24613607

[B14] Brand P. Robertson H. M. Lin W. Pothula R. Klingeman W. E. Jurat-Fuentes J. L. (2018). The origin of the odorant receptor gene family in insects. *Elife* 7:e38340. 10.7554/eLife.38340 30063003 PMC6080948

[B15] Brito N. F. Moreira M. F. Melo A. C. (2016). A look inside odorant-binding proteins in insect chemoreception. *J. Insect Physiol.* 95 51–65. 10.1016/j.jinsphys.2016.09.008 27639942

[B16] Brown A. W. Sakaria D. S. Thompson R. P. (1951). Studies on female *Aedes* mosquito. Part 1. The search for attractant vapours. *Bull. Entomol. Res.* 42 105–114. 10.1017/S0007485300025189

[B17] Cameron P. Hiroi M. Ngai J. Scott K. (2010). The molecular basis for water taste in *Drosophila*. *Nature* 465:91. 10.1038/nature09011 20364123 PMC2865571

[B18] Carey A. F. Wang G. Su C. Y. Zwiebel L. J. Carlson J. R. (2010). Odorant reception in the malaria mosquito *Anopheles gambiae*. *Nature* 464 66–71. 10.1038/nature08834 20130575 PMC2833235

[B19] Chaisson K. E. Hallem E. A. (2012). Chemosensory behaviors of parasites. *Trends Parasitol.* 28 427–436. 10.1016/j.pt.2012.07.004 22921895 PMC5663455

[B20] Chandrashekar J. Kuhn C. Oka Y. Yarmolinsky D. A. Hummler E. Ryba N. J. (2010). The cells and peripheral representation of sodium taste in mice. *Nature* 464:297. 10.1038/nature08783 20107438 PMC2849629

[B21] Chen Q. Man Y. Li J. Pei D. Wu W. (2017). Olfactory ionotropic receptors in mosquito *Aedes albopictus* (Diptera: Culicidae). *J. Med. Entomol.* 54 1229–1235. 10.1093/jme/tjx063 28399284

[B22] Chen R. Swale D. R. (2018). Inwardly rectifying potassium (Kir) channels represent a critical ion conductance pathway in the nervous systems of insects. *Sci. Rep.* 8:1617. 10.1038/s41598-018-20005-z 29371678 PMC5785497

[B23] Chen X. G. Jiang X. Gu J. Xu M. Wu Y. Deng Y. (2015). Genome sequence of the Asian Tiger mosquito, *Aedes albopictus*, reveals insights into its biology, genetics, and evolution. *Proc. Natl. Acad. Sci. U.S.A.* 112 E5907–E5915. 10.1073/pnas.1516410112 26483478 PMC4640774

[B24] Chen Y. Sun X. D. Herness S. (1996). Characteristics of action potentials and their underlying outward currents in rat taste receptor cells. *J. Neurophys.* 75 820–831. 10.1152/jn.1996.75.2.820 8714655

[B25] Clements A. N. (1992). *The Biology of Mosquitoes: Development, Nutrition and Reproduction*, Vol. 1 Wallingford: CAB International.

[B26] Cork A. Park K. C. (1996). Identification of electrophysiologically-active compounds for the malaria mosquito, *Anopheles gambiae*, in human sweat extracts. *Med. Vet. Entomol.* 10 269–276. 10.1111/j.1365-2915.1996.tb00742.x 8887339

[B27] Croset V. Rytz R. Cummins S. F. Budd A. Brawand D. Kaessmann H. (2010). Ancient protostome origin of chemosensory ionotropic glutamate receptors and the evolution of insect taste and olfaction. *PLoS Genet.* 6:e1001064. 10.1371/journal.pgen.1001064 20808886 PMC2924276

[B28] Damak S. Rong M. Yasumatsu K. Kokrashvili Z. Varadarajan V. Zou S. (2003). Detection of sweet and umami taste in the absence of taste receptor T1r3. *Science* 301 850–853. 10.1126/science.1087155 12869700

[B29] Das De T Thomas T. Verma S. Singla D. Chauhan C. Srivastava V. (2018). A synergistic transcriptional regulation of olfactory genes drives blood-feeding associated complex behavioral responses in the mosquito *Anopheles culicifacies*. *Front. Physiol.* 9:577. 10.3389/fphys.2018.00577 29875685 PMC5974117

[B30] Davis E. E. (1984). Development of lactic acid-receptor sensitivity and host-seeking behaviour in newly emerged female *Aedes aegypti* mosquitoes. *J. Insect Phys.* 30 211–215. 10.1016/0022-1910(84)90005-2 2519647

[B31] De Bruyne M. Foster K. Carlson J. R. (2001). Odor coding in the *Drosophila* antenna. *Neuron* 30 537–552. 10.1016/S0896-6273(01)00289-6 11395013

[B32] DeGennaro M. (2015). The mysterious multi-modal repellency of DEET. *Fly* 9 45–51. 10.1080/19336934.2015.1079360 26252744 PMC4594586

[B33] DeGennaro M. McBride C. S. Seeholzer L. Nakagawa T. Dennis E. J. Goldman C. (2013). Orco mutant mosquitoes lose strong preference for humans and are not repelled by volatile DEET. *Nature* 498:487. 10.1038/nature12206 23719379 PMC3696029

[B34] Dekel A. Pitts R. J. Yakir E. Bohbot J. D. (2016). Evolutionarily conserved odorant receptor function questions ecological context of octenol role in mosquitoes. *Sci. Rep.* 6:37330. 10.1038/srep37330 27849027 PMC5110965

[B35] Deng Y. Zhang W. Farhat K. Oberland S. Gisselmann G. Neuhaus E. M. (2011). The stimulatory Gαs protein is involved in olfactory signal transduction in *Drosophila*. *PLoS One* 6:e18605. 10.1371/journal.pone.0018605 21490930 PMC3072409

[B36] Dickens J. C. Bohbot J. D. (2013). Mini review: mode of action of mosquito repellents. *Pest Biochem. Physiol.* 106 149–155. 10.1016/j.pestbp.2013.02.006

[B37] Du E. J. Ahn T. J. Choi M. S. Kwon I. Kim H. W. Kwon J. Y. (2015). The mosquito repellent citronellal directly potentiates *Drosophila* TRPA1, facilitating feeding suppression. *Mol. Cell* 38 911–917. 10.14348/molcells.2015.0215 26447139 PMC4625073

[B38] Dvoryanchikov G. Sinclair M. S. Perea-Martinez I. Wang T. Chaudhari N. (2009). Inward rectifier channel, ROMK, is localized to the apical tips of glial-like cells in mouse taste buds. *J. Comp. Neurol.* 517 1–14. 10.1002/cne.22152 19708028 PMC3104395

[B39] Eleftherianos I. Won S. Chtarbanova S. Squiban B. Ocorr K. Bodmer R. (2011). ATP-sensitive potassium channel (KATP)–dependent regulation of cardiotropic viral infections. *Proc. Natl. Acad. Sci. U.S.A.* 108 12024–12029. 10.1073/pnas.1108926108 21719711 PMC3141999

[B40] Erdelyan C. N. G. Mahood T. H. Bader T. S. Y. Whyard S. (2012). Functional validation of the carbon dioxide receptor genes in *Aedes aegypti* mosquitoes using RNA interference. *Insect Mol. Biol.* 21 119–127. 10.1111/j.1365-2583.2011.01120.x 22122783

[B41] Eyun S. I. Soh H. Y. Posavi M. Munro J. B. Hughes D. S. Murali S. C. (2017). Evolutionary history of chemosensory-related gene families across the Arthropoda. *Mol. Biol. Evol.* 34 1838–1862. 10.1093/molbev/msx147 28460028 PMC5850775

[B42] Fan J. Francis F. Liu Y. Chen J. L. Cheng D. F. (2011). An overview of odorant-binding protein functions in insect peripheral olfactory reception. *Genet. Mol. Res.* 10 3056–3069. 10.4238/2011.December.8.2 22180039

[B43] Fleischer J. Pregitzer P. Breer H. Krieger J. (2018). Access to the odor world: olfactory receptors and their role for signal transduction in insects. *Cell. Mol. Life Sci.* 75 485–508. 10.1007/s00018-017-2627-5 28828501 PMC11105692

[B44] Fowler M. A. Montell C. (2013). *Drosophila* TRP channels and animal behavior. *Life Sci.* 92 394–403. 10.1016/j.lfs.2012.07.029 22877650 PMC3524398

[B45] Fox A. N. Pitts R. J. Robertson H. M. Carlson J. R. Zwiebel L. J. (2001). Candidate odorant receptors from the malaria vector mosquito *Anopheles gambiae* and evidence of down-regulation in response to blood feeding. *Proc. Natl. Acad. Sci. U.S.A.* 98 14693–14697. 10.1073/pnas.261432998 11724964 PMC64743

[B46] Freeman E. G. Dahanukar A. (2015). Molecular neurobiology of *Drosophila* taste. *Curr. Opin. Neurobiol.* 34 140–148. 10.1016/j.conb.2015.06.001 26102453 PMC4577450

[B47] Freeman E. G. Wisotsky Z. Dahanukar A. (2014). Detection of sweet tastants by a conserved group of insect gustatory receptors. *Proc. Natl. Acad. Sci. U.S.A.* 111 1598–1603. 10.1073/pnas.1311724111 24474785 PMC3910600

[B48] Fujii S. Yavuz A. Slone J. Jagge C. Song X. Amrein H. (2015). *Drosophila* sugar receptors in sweet taste perception, olfaction, and internal nutrient sensing. *Curr. Biol.* 25 621–627. 10.1016/j.cub.2014.12.058 25702577 PMC4711800

[B49] Fuller J. L. (1974). Single-locus control of saccharin preference in mice. *J. Hered.* 65 33–36. 10.1093/oxfordjournals.jhered.a108452 4847746

[B50] Gilbertson T. A. (1993). The physiology of vertebrate taste reception. *Curr. Opin. Neurobiol.* 3 532–539. 10.1016/0959-4388(93)90052-Z 7693091

[B51] Gilbertson T. A. Fontenot D. T. Liu L. Zhang H. Monroe W. T. (1997). Fatty acid modulation of K+ channels in taste receptor cells: gustatory cues for dietary fat. *Am. J. Physiol. – Cell Physiol.* 272 C1203–C1210. 10.1152/ajpcell.1997.272.4.C1203 9142845

[B52] Hallem E. A. Fox A. N. Zwiebel L. J. Carlson J. R. (2004). Olfaction: mosquito receptor for human-sweat odorant. *Nature* 427:212. 10.1038/427212a 14724626

[B53] Herness M. S. Sun X. D. (1995). Voltage-dependent sodium currents recorded from dissociated rat taste cells. *J. Membr. Biol.* 146 73–84. 10.1007/BF00232681 7563038

[B54] Herness M. S. Sun X. D. (1999). Characterization of chloride currents and their noradrenergic modulation in rat taste receptor cells. *J. Neurophysiol.* 82 260–271. 10.1152/jn.1999.82.1.260 10400955

[B55] Hibino H. Inanobe A. Furutani K. Murakami S. Findlay I. Kurachi Y. (2010). Inwardly rectifying potassium channels: their structure, function, and physiological roles. *Physiol. Rev.* 90 291–366. 10.1152/physrev.00021.2009 20086079

[B56] Hill C. A. Fox A. N. Pitts R. J. Kent L. B. Tan P. L. Chrystal M. A. (2002). G protein-coupled receptors in *Anopheles gambiae*. *Science* 298 176–178. 10.1126/science.1076196 12364795

[B57] Hill S. R. Majeed S. Ignell R. (2015). Molecular basis for odorant receptor tuning: a short C-terminal sequence is necessary and sufficient for selectivity of mosquito Or8. *Insect Mol. Biol.* 24 491–501. 10.1111/imb.12176 26033210

[B58] Huang L. Cao J. Wang H. Vo L. A. Brand J. G. (2005). Identification and functional characterization of a voltage-gated chloride channel and its novel splice variant in taste bud cells. *J. Biol. Chem.* 280 36150–36157. 10.1074/jbc.M507706200 16129671 PMC2367165

[B59] Hughes D. T. Pelletier J. Luetje C. W. Leal W. S. (2010). Odorant receptor from the southern house mosquito narrowly tuned to the oviposition attractant skatole. *J. Chem. Ecol.* 36 797–800. 10.1007/s10886-010-9828-9 20623327 PMC2908433

[B60] Ignell R. Hansson B. S. (2005). Projection patterns of gustatory neurons in the suboesophageal ganglion and tritocerebrum of mosquitoes. *J. Comp. Neurol.* 492 214–233. 10.1002/cne.20691 16196031

[B61] Ishimoto H. Takahashi K. Ueda R. Tanimura T. (2005). G-protein gamma subunit 1 is required for sugar reception in *Drosophila*. *EMBO J.* 24 3259–3265. 10.1038/sj.emboj.7600796 16121192 PMC1224686

[B62] Isono K. Morita H. (2010). Molecular and cellular designs of insect taste receptor system. *Front. Cell. Neurosci.* 4:20. 10.3389/fncel.2010.00020 20617187 PMC2896210

[B63] Jeong Y. T. Shim J. Oh S. R. Yoon H. I. Kim C. H. Moon S. J. (2013). An odorant-binding protein required for the suppression of sweet taste by bitter chemicals. *Neuron* 79 725–737. 10.1016/j.neuron.2013.06.025 23972598 PMC3753695

[B64] Jones W. D. Nguyen T. A. T. Kloss B. Lee K. J. Vosshall L. B. (2005). Functional conservation of an insect odorant receptor gene across 250 million years of evolution. *Curr. Biol.* 15 R119–R121. 10.1016/j.cub.2005.02.007 15723778

[B65] Kang K. Pulver S. R. Panzano V. C. Chang E. C. Griffith L. C. Theobald D. L. (2010). Analysis of *Drosophila* TRPA1 reveals an ancient origin of human chemical nociception. *Nature* 464 597–600. 10.1038/nature08848 20237474 PMC2845738

[B66] Kent L. B. Walden K. K. Robertson H. M. (2008). The Gr family of candidate gustatory and olfactory receptors in the yellow-fever mosquito *Aedes aegypti*. *Chem. Senses* 33 79–93. 10.1093/chemse/bjm067 17928357

[B67] Kessler S. Vlimant M. Guerin P. M. (2013). The sugar meal of the African malaria mosquito *Anopheles gambiae* and how deterrent compounds interfere with it: a behavioural and neurophysiological study. *J. Exp. Biol.* 216 1292–1306. 10.1242/jeb.076588 23264482

[B68] Kim S. H. Lee Y. Akitake B. Woodward O. M. Guggino W. B. Montell C. (2010). *Drosophila* TRPA1 channel mediates chemical avoidance in gustatory receptor neurons. *Proc. Natl. Acad. Sci. U.S.A.* 107 8440–8445. 10.1073/pnas.1001425107 20404155 PMC2889570

[B69] Kinnamon S. C. (1992). “Role of K channels in taste transduction,” in *Sensory Transduction*, eds Corey D. P. Roper S. D. (New York, NY: Rockefeller University Press), 261–270. 1369766

[B70] Kofuji P. Newman E. A. (2004). Potassium buffering in the central nervous system. *Neuroscience* 129 1043–1054. 10.1016/j.neuroscience.2004.06.008 15561419 PMC2322935

[B71] Kretz O. Barbry P. Bock R. Lindemann B. (1999). Differential expression of RNA and protein of the three pore-forming subunits of the amiloride-sensitive epithelial sodium channel in taste buds of the rat. *J. Histochem. Cytochem.* 47 51–64. 10.1177/002215549904700106 9857212

[B72] Kwon H. W. Lu T. Rützler M. Zwiebel L. J. (2006). Olfactory responses in a gustatory organ of the malaria vector mosquito *Anopheles gambiae*. *Proc. Natl. Acad. Sci. U.S.A.* 103 13526–13531. 10.1073/pnas.0601107103 16938890 PMC1569196

[B73] Kwon Y. Kim S. H. Ronderos D. S. Lee Y. Akitake B. Woodward O. M. (2010). *Drosophila* TRPA1 channel is required to avoid the naturally occurring insect repellent citronellal. *Curr. Biol.* 20 1672–1678. 10.1016/j.cub.2010.08.016 20797863 PMC2946437

[B74] Larsson M. C. Domingos A. I. Jones W. D. Chiappe M. E. Amrein H. Vosshall L. B. (2004). Or83b encodes a broadly expressed odorant receptor essential for *Drosophila* olfaction. *Neuron* 43 703–714. 10.1016/j.neuron.2004.08.019 15339651

[B75] Latorre R. Miller C. (1983). Conduction and selectivity in potassium channels. *J. Membr. Biol.* 71 11–30. 10.1007/BF01870671 6300405

[B76] Leal W. S. (2013). Odorant reception in insects: roles of receptors, binding proteins, and degrading enzymes. *Ann. Rev. Entomol.* 58 373–391. 10.1146/annurev-ento-120811-153635 23020622

[B77] Lee Y. Kim S. H. Montell C. (2010). Avoiding DEET through insect gustatory receptors. *Neuron* 67 555–561. 10.1016/j.neuron.2010.07.006 20797533 PMC2929391

[B78] Lindemann B. (1997). Sodium taste. *Curr. Opin. Nephrol. Hypertens.* 6 425–429. 10.1097/00041552-199709000-00003 9327199

[B79] Lindemann B. Barbry P. Kretz O. Bock R. (1998). Occurrence of ENaC subunit mRNA and immunocytochemistry of the channel subunits in taste buds of the rat vallate papilla. *Ann. N. Y. Acad. Sci.* 855 116–127. 10.1111/j.1749-6632.1998.tb10553.x 9929592

[B80] Liu C. Pitts R. Bohbot J. D. Jones P. L. Wang G. Zwiebel L. J. (2010). Distinct olfactory signaling mechanisms in the malaria vector mosquito *Anopheles gambiae*. *PLoS Biol.* 8:e1000467. 10.1371/journal.pbio.1000467 20824161 PMC2930861

[B81] Liu L. Leonard A. S. Motto D. G. Feller M. A. Price M. P. Johnson W. A. (2003). Contribution of *Drosophila* DEG/ENaC genes to salt taste. *Neuron* 39 133–146. 10.1016/S0896-6273(03)00394-5 12848938

[B82] Logan J. G. Birkett M. A. Clark S. J. Powers S. Seal N. J. Wadhams L. J. (2008). Identification of human-derived volatile chemicals that interfere with attraction of *Aedes aegypti* mosquitoes. *J. Chem. Ecol.* 34:308. 10.1007/s10886-008-9436-0 18306972

[B83] Lombardo F. Salvemini M. Fiorillo C. Nolan T. Zwiebel L. J. Ribeiro J. M. (2017). Deciphering the olfactory repertoire of the tiger mosquito *Aedes albopictus*. *BMC Genomics* 18:770. 10.1186/s12864-017-4144-1 29020917 PMC5637092

[B84] Lu T. Qiu Y. T. Wang G. Kwon J. Y. Rutzler M. Kwon H. W. (2007). Odor coding in the maxillary palp of the malaria vector mosquito *Anopheles gambiae*. *Curr. Biol.* 17 1533–1544. 10.1016/j.cub.2007.07.062 17764944 PMC3113458

[B85] Matsuura H. Sokabe T. Kohno K. Tominaga M. Kadowaki T. (2009). Evolutionary conservation and changes in insect TRP channels. *BMC Evol. Biol.* 9:228. 10.1186/1471-2148-9-228 19740447 PMC2753570

[B86] Matthews B. J. McBride C. S. DeGennaro M. Despo O. Vosshall L. B. (2016). The neurotranscriptome of the *Aedes aegypti* mosquito. *BMC Genomics* 17:32. 10.1186/s12864-015-2239-0 26738925 PMC4704297

[B87] McBride C. S. Baier F. Omondi A. B. Spitzer S. A. Lutomiah J. Sang R. (2014). Evolution of mosquito preference for humans linked to an odorant receptor. *Nature* 515 222–227. 10.1038/nature13964 25391959 PMC4286346

[B88] McIver S. B. (1982). Review article: sensillae of mosquitoes. *J. Med. Entomol.* 19 489–535. 6128422 10.1093/jmedent/19.5.489

[B89] McMeniman C. J. Corfas R. A. Matthews B. J. Ritchie S. A. Vosshall L. B. (2014). Multimodal integration of carbon dioxide and other sensory cues drives mosquito attraction to humans. *Cell* 156 1060–1071. 10.1016/j.cell.2013.12.044 24581501 PMC4007582

[B90] Meijerink J. Braks M. A. H. Van Loon J. J. A. (2001). Olfactory receptors on the antennae of the malaria mosquito *Anopheles gambiae* are sensitive to ammonia and other sweat-borne components. *J. Insect Physiol.* 47 455–464. 10.1016/S0022-1910(00)00136-0 11166310

[B91] Melo A. C. A. Rützler M. Pitts R. J. Zwiebel L. J. (2004). Identification of a chemosensory receptor from the yellow fever mosquito. *Aedes aegypti*, that is highly conserved and expressed in olfactory and gustatory organs. *Chem. Senses* 29 403–410. 10.1093/chemse/bjh041 15201207

[B92] Missbach C. Dweck H. K. Vogel H. Vilcinskas A. Stensmyr M. C. Hansson B. S. (2014). Evolution of insect olfactory receptors. *Elife* 3:e02115. 10.7554/eLife.02115 24670956 PMC3966513

[B93] Miyamoto T. Fujiyama R. Okada Y. Sato T. (1998). Sour transduction involves activation of NPPB-sensitive conductance in mouse taste cells. *J. Neurophysiol.* 80 1852–1859. 10.1152/jn.1998.80.4.1852 9772244

[B94] Nakagawa T. Pellegrino M. Sato K. Vosshall L. B. Touhara K. (2012). Amino acid residues contributing to function of the heteromeric insect olfactory receptor complex. *PLoS One* 7:e32372. 10.1371/journal.pone.0032372 22403649 PMC3293798

[B95] Neusch C. Papadopoulos N. Muller M. Maletzki I. Winter S. M. Hirrlinger J. (2006). Lack of the Kir4. 1 channel subunit abolishes K+ buffering properties of astrocytes in the ventral respiratory group: impact on extracellular K+ regulation. *J. Neurophysiol.* 95 1843–1852. 10.1152/jn.00996.2005 16306174

[B96] Nichols A. S. Chen S. Luetje C. W. (2011). Subunit contributions to insect olfactory receptor function: channel block and odorant recognition. *Chem. Senses* 36 781–790. 10.1093/chemse/bjr053 21677030 PMC3195787

[B97] Nilius B. (2003). From TRPs to SOCs, CCEs, and CRACs: consensus and controversies. *Cell Calcium* 33 293–298. 10.1016/S0143-4160(03)00042-3 12765675

[B98] Nolte A. Gawalek P. Koerte S. Wei H. Schumann R. Werckenthin A. (2016). No evidence for ionotropic pheromone transduction in the hawkmoth *Manduca sexta*. *PLoS One* 11:e0166060. 10.1371/journal.pone.0166060 27829053 PMC5102459

[B99] Ohmoto M. Matsumoto I. Misaka T. Abe K. (2006). Taste receptor cells express voltage-dependent potassium channels in a cell age–specific manner. *Chem. Senses* 31 739–746. 10.1093/chemse/bjl016 16873422

[B100] O’Neal S. T. Swale D. R. Anderson T. D. (2017a). ATP-sensitive inwardly rectifying potassium channel regulation of viral infections in honey bees. *Sci. Rep.* 7:8668. 10.1038/s41598-017-09448-y 28819165 PMC5561242

[B101] O’Neal S. T. Swale D. R. Bloomquist J. R. Anderson T. D. (2017b). ATP-sensitive inwardly rectifying potassium channel modulators alter cardiac function in honey bees. *J. Insect Physiol.* 99 95–100. 10.1016/j.jinsphys.2017.04.005 28412203

[B102] Pappas L. G. Larsen J. R. (1978). Gustatory mechanisms and sugar-feeding in the mosquito *Culiseta inornata*. *Physiol. Entomol.* 3 115–119. 10.1111/j.1365-3032.1978.tb00141.x

[B103] Pelletier J. Hughes D. T. Luetje C. W. Leal W. S. (2010). An odorant receptor from the southern house mosquito *Culex pipiens* quinquefasciatus sensitive to oviposition attractants. *PLoS One* 5:e10090. 10.1371/journal.pone.0010090 20386699 PMC2851645

[B104] Pelosi P. Iovinella I. Zhu J. Wang G. Dani F. R. (2018). Beyond chemoreception: diverse tasks of soluble olfactory proteins in insects. *Biol. Rev.* 93 184–200. 10.1111/brv.12339 28480618

[B105] Pitts R. J. Derryberry S. L. Zhang Z. Zwiebel L. J. (2017). Variant ionotropic receptors in the malaria vector mosquito *Anopheles gambiae* tuned to amines and carboxylic acids. *Sci. Rep.* 7:40297. 10.1038/srep40297 28067294 PMC5220300

[B106] Pitts R. J. Fox A. N. Zwiebel L. J. (2004). A highly conserved candidate chemoreceptor expressed in both olfactory and gustatory tissues in the malaria vector *Anopheles gambiae*. *Proc. Natl. Acad. Sci. U.S.A.* 101 5058–5063. 10.1073/pnas.0308146101 15037749 PMC387373

[B107] Pitts R. J. Rinker D. C. Jones P. L. Rokas A. Zwiebel L. J. (2011). Transcriptome profiling of chemosensory appendages in the malaria vector *Anopheles gambiae* reveals tissue- and sex-specific signatures of odor coding. *BMC Genomics* 12:271. 10.1186/1471-2164-12-271 21619637 PMC3126782

[B108] Purves D. Augustine G. J. Fitzpatrick D. Katz L. C. LaMantia A. S. McNamara J. O., eds (2001). *Neuroscience*, 2nd Edn. Sunderland, MA: Sinauer Associates.

[B109] Qiu Y. T. Smallegange R. C. Hoppe S. Van Loon J. J. A. Bakker E. J. Takken W. (2004). Behavioural and electrophysiological responses of the malaria mosquito *Anopheles gambiae* Giles sensu stricto (Diptera: Culicidae) to human skin emanations. *Med. Vet. Entomol.* 18 429–438. 10.1111/j.0269-283X.2004.00534.x 15642010

[B110] Riabinina O. Task D. Marr E. Lin C. Alford R. O’Brochta D. A. (2016). Organization of olfactory centres in the malaria mosquito *Anopheles gambiae*. *Nat. Comm.* 7:13010. 10.1038/ncomms13010 27694947 PMC5063964

[B111] Rinker D. C. Pitts R. J. Zhou X. Suh E. Rokas A. Zwiebel L. J. (2013a). Blood meal-induced changes to antennal transcriptome profiles reveal shifts in odor sensitivities in *Anopheles gambiae*. *Proc. Natl. Acad. Sci. U.S.A.* 110 8260–8265. 10.1073/pnas.1302562110 23630291 PMC3657813

[B112] Rinker D. C. Zhou X. Pitts R. J. Rokas A. Zwiebel L. J. (2013b). Antennal transcriptome profiles of anopheline mosquitoes reveal human host olfactory specialization in *Anopheles gambiae*. *BMC Genomics* 14:749. 10.1186/1471-2164-14-749 24182346 PMC3833343

[B113] Robertson H. M. (2015). The insect chemoreceptor superfamily is ancient in animals. *Chem. Senses* 40 609–614. 10.1093/chemse/bjv046 26354932

[B114] Robertson H. M. Warr C. G. Carlson J. R. (2003). Molecular evolution of the insect chemoreceptor gene superfamily in *Drosophila melanogaster*. *Proc. Natl. Acad. Sci. U.S.A.* 100 14537–14542. 10.1073/pnas.2335847100 14608037 PMC304115

[B115] Rosenzweig M. Brennan K. M. Tayler T. D. Phelps P. O. Patapoutian A. Garrity P. A. (2005). The *Drosophila* ortholog of vertebrate TRPA1 regulates thermotaxis. *Genes Dev.* 19 419–424. 10.1101/gad.1278205 15681611 PMC548941

[B116] Rudy B. (1988). Diversity and ubiquity of K channels. *Neuroscience* 25 729–749. 10.1016/0306-4522(88)90033-4 2457185

[B117] Rund S. S. Bonar N. A. Champion M. M. Ghazi J. P. Houk C. M. Leming M. T. (2013). Daily rhythms in antennal protein and olfactory sensitivity in the malaria mosquito *Anopheles gambiae*. *Sci. Rep.* 3:2494. 10.1038/srep02494 23986098 PMC3756343

[B118] Rytz R. Croset V. Benton R. (2013). Ionotropic receptors (IRs): chemosensory ionotropic glutamate receptors in *Drosophila* and beyond. *Insect. Biochem. Mol. Biol.* 43 888–897. 10.1016/j.ibmb.2013.02.007 23459169

[B119] Sanford J. L. Shields V. D. C. Dickens J. C. (2013). Gustatory receptor neuron responds to DEET and other insect repellents in the yellow-fever mosquito, *Aedes aegypti. Naturwiss* 100 269–273. 10.1007/s00114-013-1021-x 23407786

[B120] Sato K. Pellegrino M. Nakagawa T. Nakagawa T. Vosshall L. B. Touhara K. (2008). Insect olfactory receptors are heteromeric ligand-gated ion channels. *Nature* 452 1002–1006. 10.1038/nature06850 18408712

[B121] Sato K. Tanaka K. Touhara K. (2011). Sugar-regulated cation channel formed by an insect gustatory receptor. *Proc. Natl. Acad. Sci. U.S.A.* 108 11680–11685. 10.1073/pnas.1019622108 21709218 PMC3136286

[B122] Siju K. P. Hansson B. S. Ignell R. (2008). Immunocytochemical localization of serotonin in the central and peripheral chemosensory system of mosquitoes. *Arthropod Struct. Dev.* 37 248–259. 10.1016/j.asd.2007.12.001 18424232

[B123] Sim S. Ramirez J. L. Dimopoulos G. (2012). Dengue virus infection of the *Aedes aegypti* salivary gland and chemosensory apparatus induces genes that modulate infection and blood-feeding behavior. *PLoS Pathog.* 8:e1002631. 10.1371/journal.ppat.1002631 22479185 PMC3315490

[B124] Slifer E. H. (1962). Sensory hairs with permeable tips on the tarsi of the yellow-fever mosquito, *Aedes aegypti. Ann. Entomol. Soc. Am.* 55 531–535. 10.1093/aesa/55.5.531

[B125] Soldano A. Alpizar Y. A. Boonen B. Franco L. Lopez-Requena A. Liu G. (2016). Gustatory-mediated avoidance of bacterial lipopolysaccharides via TRPA1 activation in *Drosophila*. *Elife* 5:e13133. 10.7554/eLife.13133 27296646 PMC4907694

[B126] Sparks J. T. Bohbot J. D. Dickens J. C. (2014). The genetics of chemoreception in the labella and tarsi of *Aedes aegypti*. *Insect Biochem. Mol. Biol.* 48 8–16. 10.1016/j.ibmb.2014.02.004 24582661

[B127] Sparks J. T. Dickens J. C. (2016). Bitter-sensitive gustatory receptor neuron responds to chemically diverse insect repellents in the common malaria mosquito *Anopheles quadrimaculatus*. *Sci. Nat.* 103:39. 10.1007/s00114-016-1367-y 27108454

[B128] Sparks J. T. Vinyard B. T. Dickens J. C. (2013). Gustatory receptor expression in the labella and tarsi of *Aedes aegypti*. *Insect Biochem. Mol. Biol.* 43 1161–1171. 10.1016/j.ibmb.2013.10.005 24157615

[B129] Suh E. Bohbot J. D. Zwiebel L. J. (2014). Peripheral olfactory signaling in insects. *Curr. Opin. Insect Sci.* 6 86–92. 10.1016/j.cois.2014.10.006 25584200 PMC4288021

[B130] Sun X. D. Herness M. S. (1996). Characterization of inwardly rectifying potassium currents from dissociated rat taste receptor cells. *Am. J. Physiol. – Cell Physiol.* 271 C1221–C1232. 10.1152/ajpcell.1996.271.4.C1221 8897828

[B131] Survery S. Moparthi L. Kjellbom P. Högestätt E. D. Zygmunt P. M. Johanson U. (2016). The N-terminal ankyrin repeat domain is not required for electrophile and heat activation of the purified mosquito TRPA1 receptor. *J. Biol. Chem.* 291 26899–26912. 10.1074/jbc.M116.743443 27875296 PMC5207195

[B132] Swale D. R. Li Z. Guerrero F. De León A. A. P. Foil L. D. (2017). Role of inward rectifier potassium channels in salivary gland function and sugar feeding of the fruit fly, *Drosophila melanogaster. Pestic. Biochem. Physiol.* 141 41–49. 10.1016/j.pestbp.2016.11.005 28911739

[B133] Ueno K. Kidokoro Y. (2008). Adenylyl cyclase encoded by AC78C participates in sugar perception in Drosophila melanogaster. *Eur. J. Neurosci.* 28 1956–1966. 10.1111/j.1460-9568.2008.06507.x 19046378

[B134] Ueno K. Kohatsu S. Clay C. Forte M. Isono K. Kidokoro Y. (2006). Gsα is involved in sugar perception in *Drosophila melanogaster*. *J. Neurosci.* 26 6143–6152. 10.1523/JNEUROSCI.0857-06.2006 16763022 PMC6675175

[B135] Urrego D. Tomczak A. P. Zahed F. Stühmer W. Pardo L. A. (2014). Potassium channels in cell cycle and cell proliferation. *Philos. Trans. R. Soc. B* 369:20130094. 10.1098/rstb.2013.0094 24493742 PMC3917348

[B136] Van der Goes van Naters W. Carlson J. R. (2006). Insects as chemosensors of humans and crops. *Nature* 444 302–307. 10.1038/nature05403 17108954

[B137] Van Giesen L. Hernandez-Nunez L. Delasoie-Baranek S. Colombo M. Renaud P. Bruggmann R. (2016). Multimodal stimulus coding by a gustatory sensory neuron in *Drosophila larvae*. *Nat. Comm.* 7:10687. 10.1038/ncomms10687 26864722 PMC4753250

[B138] Vannier B. Zhu X. Brown D. Birnbaumer L. (1998). The membrane topology of human transient receptor potential 3 as inferred from glycosylation-scanning mutagenesis and epitope immunocytochemistry. *J. Biol. Chem.* 273 8675–8679. 10.1074/jbc.273.15.8675 9535843

[B139] Venkatachalam K. Montell C. (2007). TRP channels. *Annu. Rev. Biochem.* 76 387–417. 10.1146/annurev.biochem.75.103004.142819 17579562 PMC4196875

[B140] Vogt R. G. Callahan F. E. Rogers M. E. Dickens J. C. (1999). Odorant binding protein diversity and distribution among the insect orders, as indicated by LAP, an OBP-related protein of the true bug *Lygus lineolaris* (Hemiptera, Heteroptera). *Chem. Senses* 24 481–495. 10.1093/chemse/24.5.481 10576256

[B141] Vogt R. G. Riddiford L. M. (1981). Pheromone binding and inactivation by moth antennae. *Nature* 29 161–163. 10.1038/293161a0 18074618

[B142] Wang G. Carey A. F. Carlson J. R. Zwiebel L. J. (2010). Molecular basis of odor coding in the malaria vector mosquito *Anopheles gambiae*. *Proc. Natl. Acad. Sci. U.S.A.* 107 4418–4423. 10.1073/pnas.0913392107 20160092 PMC2840125

[B143] Wang G. Qiu Y. T. Lu T. Kwon H. W. Pitts R. J. Van Loon J. J. (2009). *Anopheles gambiae* TRPA1 is a heat-activated channel expressed in thermosensitive sensilla of female antennae. *Eur. J. Neurosci.* 30 967–974. 10.1111/j.1460-9568.2009.06901.x 19735290 PMC3106298

[B144] Watson K. J. Kim I. Baquero A. F. Burks C. A. Liu L. Gilbertson T. A. (2007). Expression of aquaporin water channels in rat taste buds. *Chem. Senses* 32 411–421. 10.1093/chemse/bjm006 17339611

[B145] Weaver S. C. Reisen W. K. (2010). Present and future arboviral threats. *Antiviral Res.* 85 328–345. 10.1016/j.antiviral.2009.10.008 19857523 PMC2815176

[B146] Wicher D. (2015). “Olfactory signaling in insects,” in *Progress in Molecular Biology and Translational Science*, ed. Glatz R. (Waltham, MA: Academic Press),37–54. 10.1016/bs.pmbts.2014.11.002 25623336

[B147] Wicher D. Schäfer R. Bauernfeind R. Stensmyr M. C. Heller R. Heinemann S. H. (2008). *Drosophila* odorant receptors are both ligand-gated and cyclic-nucleotide-activated cation channels. *Nature* 452 1007–1011. 10.1038/nature06861 18408711

[B148] World Health Organization [WHO]. (2017). *World Malaria Report 2017.* Geneva: WHO 10.30875/50d27d62-en

[B149] Xia Y. Wang G. Buscariollo D. Pitts R. J. Wenger H. Zwiebel L. J. (2008). The molecular and cellular basis of olfactory-driven behavior in *Anopheles gambiae* larvae. *Proc. Natl. Acad. Sci. U.S.A.* 105 6433–6438. 10.1073/pnas.0801007105 18427108 PMC2359781

[B150] Xu P. Choo Y. M. De La Rosa A. Leal W. S. (2014). Mosquito odorant receptor for DEET and methyl jasmonate. *Proc. Natl. Acad. Sci. U.S.A.* 111 16592–16597. 10.1073/pnas.1417244111 25349401 PMC4246313

[B151] Yao C. A. Ignell R. Carlson J. R. (2005). Chemosensory coding by neurons in the coeloconic sensilla of the *Drosophila* antenna. *J. Neurosci.* 25 8359–8367. 10.1523/JNEUROSCI.2432-05.2005 16162917 PMC6725686

[B152] Yarmolinsky D. A. Zuker C. S. Ryba N. J. (2009). Common sense about taste: from mammals to insects. *Cell* 139 234–244. 10.1016/j.cell.2009.10.001 19837029 PMC3936514

[B153] Ye W. Chang R. B. Bushman J. D. Tu Y. H. Mulhall E. M. Wilson C. E. (2016). The K+ channel KIR2.1 functions in tandem with proton influx to mediate sour taste transduction. *Proc. Natl. Acad. Sci. U.S.A.* 113 E229–E238. 10.1073/pnas.1514282112 26627720 PMC4720319

[B154] Yee K. K. Sukumaran S. K. Kotha R. Gilbertson T. A. Margolskee R. F. (2011). Glucose transporters and ATP-gated K+ (KATP) metabolic sensors are present in type 1 taste receptor 3 (T1r3)-expressing taste cells. *Proc. Natl. Acad. Sci. U.S.A.* 108 5431–5436. 10.1073/pnas.1100495108 21383163 PMC3069197

[B155] Zars T. (2000). Behavioral functions of the insect mushroom bodies. *Curr. Opin. Neurobiol.* 10 790–795. 10.1016/S0959-4388(00)00147-1 11240291

[B156] Zelle K. M. Lu B. Pyfrom S. C. Ben-Shahar Y. (2013). The genetic architecture of degenerin/epithelial sodium channels in *Drosophila*. *G3 (Bethesda)* 3 441–450. 10.1534/g3.112.005272 23449991 PMC3583452

[B157] Zhang H. J. Anderson A. R. Trowell S. C. Luo A. R. Xiang Z. H. Xia Q. Y. (2011). Topological and functional characterization of an insect gustatory receptor. *PLoS One* 6:e24111. 10.1371/journal.pone.0024111 21912618 PMC3163651

[B158] Zhang Y. V. Raghuwanshi R. P. Shen W. L. Montell C. (2013). Food experience–induced taste desensitization modulated by the *Drosophila* TRPL channel. *Nat. Neurosci.* 16:1468. 10.1038/nn.3513 24013593 PMC3785572

